# Phenotype Instance Verification and Evaluation Tool (PIVET): A Scaled Phenotype Evidence Generation Framework Using Web-Based Medical Literature

**DOI:** 10.2196/jmir.9610

**Published:** 2018-05-04

**Authors:** Jette Henderson, Junyuan Ke, Joyce C Ho, Joydeep Ghosh, Byron C Wallace

**Affiliations:** ^1^ The University of Texas at Austin Austin, TX United States; ^2^ Emory University Atlanda, GA United States; ^3^ Northeastern University Boston, MA United States

**Keywords:** medical informatics, medical subject headings, algorithms, clustering analysis, classification, databases as topic, information storage and retrieval, MEDLINE

## Abstract

**Background:**

Researchers are developing methods to automatically extract clinically relevant and useful patient characteristics from raw healthcare datasets. These characteristics, often capturing essential properties of patients with common medical conditions, are called *computational phenotypes.* Being generated by automated or semiautomated, data-driven methods, such potential phenotypes need to be validated as clinically meaningful (or not) before they are acceptable for use in decision making.

**Objective:**

The objective of this study was to present Phenotype Instance Verification and Evaluation Tool (PIVET), a framework that uses co-occurrence analysis on an online corpus of publically available medical journal articles to build clinical relevance evidence sets for user-supplied phenotypes. PIVET adopts a conceptual framework similar to the pioneering prototype tool PheKnow-Cloud that was developed for the phenotype validation task. PIVET completely refactors each part of the PheKnow-Cloud pipeline to deliver vast improvements in speed without sacrificing the quality of the insights PheKnow-Cloud achieved.

**Methods:**

PIVET leverages indexing in NoSQL databases to efficiently generate evidence sets. Specifically, PIVET uses a succinct representation of the phenotypes that corresponds to the index on the corpus database and an optimized co-occurrence algorithm inspired by the Aho-Corasick algorithm. We compare PIVET’s phenotype representation with PheKnow-Cloud’s by using PheKnow-Cloud’s experimental setup. In PIVET’s framework, we also introduce a statistical model trained on domain expert–verified phenotypes to automatically classify phenotypes as clinically relevant or not. Additionally, we show how the classification model can be used to examine user-supplied phenotypes in an online, rather than batch, manner.

**Results:**

PIVET maintains the discriminative power of PheKnow-Cloud in terms of identifying clinically relevant phenotypes for the same corpus with which PheKnow-Cloud was originally developed, but PIVET’s analysis is an order of magnitude faster than that of PheKnow-Cloud. Not only is PIVET much faster, it can be scaled to a larger corpus and still retain speed. We evaluated multiple classification models on top of the PIVET framework and found ridge regression to perform best, realizing an average F1 score of 0.91 when predicting clinically relevant phenotypes.

**Conclusions:**

Our study shows that PIVET improves on the most notable existing computational tool for phenotype validation in terms of speed and automation and is comparable in terms of accuracy.

## Introduction

### Computational Phenotyping

The rapidly expanding availability of electronic health records (EHRs) offers the promise to help clinicians better understand the populations they serve. The ability to efficiently characterize large volumes of healthcare data is essential to enabling clinicians to use this information effectively. Recently, machine learning and data mining researchers have attempted to address this need in several ways. One such line of work concerns developing methods to extract computational phenotypes from raw health data in an automated, high-throughput manner. Here we define a computational phenotype as a constellation of clinically interesting characteristics that delineates a cohesive group of patients. Such phenotypes can help clinicians reason about patient populations, identify patient cohorts, and identify and describe the progression of diseases within populations.

Although being able to extract phenotypes in a high-throughput manner constitutes a potentially important step in helping clinicians reason about their patient populations on a larger scale, this potential will be realized only if the identified phenotypes are clinically meaningful. Therefore, to increase the utility of data-driven phenotypes, some measure quantifying the inferred clinical meaningfulness should be reported alongside the phenotypes to help practitioners sort signal from noise. To address this need, we present Phenotype Instance Verification and Evaluation Tool (PIVET), a tool that uses analysis of open access (OA) PubMed (a corpus of online medical articles) to generate evidence sets and clinical relevance scores for candidate phenotypes. These evidence sets can be used by researchers when developing and tuning new computational phenotype methods; domain experts when they are validating candidate phenotypes; and eventually, clinicians examining the phenotypes associated with their patient populations.

PIVET is an improvement on a recently introduced prototype tool called PheKnow-Cloud [1]. PheKnow-Cloud, which earned the Distinguished Paper Award at the 2017 AMIA Joint Summits, demonstrated that the medical expertise contained in PubMed articles could be harnessed to build evidence sets for the clinical validity of candidate phenotypes. PIVET is built on the same conceptual framework as PheKnow-Cloud, but in PIVET, we have optimized each piece of PheKnow-Cloud’s pipeline to deliver vast improvements in speed and interpretability without sacrificing the integrity of PheKnow-Cloud’s phenotype evaluation.

The PheKnow-Cloud pipeline consists of three major steps: (1) representing each phenotype so occurrences of it and related terms in the corpus will be recognized (phenotypic representation), (2) analyzing the corpus using the phenotype representation (corpus analysis), and (3) calculating a clinical relevance score and designation (clinical validity determination). In the phenotype representation step, PIVET uses succinct and possibly more interpretable representations of terms contained within each phenotype. In the corpus analysis step, PIVET migrates from a brute force approach of analyzing the corpus to an approach that uses a NoSQL database to store and index the articles efficiently. PIVET then utilizes a variation of the Aho-Corasick algorithm to count appearances of the terms within each phenotype. Finally, in the clinical validity calculation step, PIVET streamlines the clinical relevance score analysis and uses a model, trained on domain expert–verified phenotypes, to classify the clinical relevance of supplied phenotypes. Through a combination of these improvements, PIVET runs an order of magnitude faster than PheKnow-Cloud without sacrificing the discriminative power of the original tool.

PheKnow-Cloud was developed to function in high-throughput phenotyping situations where a researcher has a large set of potential phenotypes to validate. Consequently, PheKnow-Cloud was built to run only in a batch setting. However, in clinical settings and some research settings, a user may only have a few new phenotypes to analyze, so we developed PIVET to run in either an online or batch environment. This improvement will allow clinicians to query PIVET even with single phenotypes, which could possibly help in decision-making processes. Additionally, it could help researchers tune their phenotype extraction algorithms. Thus, while the prototype tool demonstrated the analysis of medical articles could be used to evaluate candidate phenotypes, the improvements in speed and automation realized by PIVET make it useful in both research and clinical settings.

The paper is organized as follows. We first present research related to PIVET, including a description of the original prototype tool (PheKnow-Cloud). Next, we describe the PIVET framework, noting the important differences between PheKnow-Cloud and the new system. We then report the performance of PIVET on automatically generated phenotypes as well as domain expert–curated phenotypes and demonstrate how the framework can be used in an online setting. We conclude the paper with a discussion of the limitations of this work and thoughts on future directions.

### Related Work

#### PubMed

PubMed Central (PMC) is an online collection comprising over 3 million biomedical and biological articles gathered from thousands of journals [2]. PMC is maintained and curated by the National Library of Medicine (NLM) at the US National Institute of Health [3].

In regard to phenotypes, researchers tend to use PubMed as an exploratory tool to *discover* new phenotypes rather than as a resource to *validate* candidate phenotypes. Boland et al orchestrated one of the few studies that used PubMed as a validation tool. They mined EHRs for patients with predefined disease codes and then compared the birth month and the disease of these patients with a group of control patients who did not have the disease codes present in their EHRs. They found a relationship between certain diseases and birth months in the case group [4]. They validated their results against papers retrieved from PubMed that mentioned disease and birth month. This study was novel in that it demonstrated PubMed could be utilized to provide feedback for and validation of results produced through automatic means.

More commonly, researchers use PubMed as tool to generate hypotheses and discover phenotypes and other biomedical issues [5,6]. Multiple software packages such as LitInspector (Genomatix Software Suite) [7], PubMed.mineR (CSIR) [8], ALIBABA (Humboldt-Universität zu Berlin) [9], as well as python packages such as Pymedtermino (Paris 13 University) [10] and Biopython (Open Bioinformatics Foundation) [11] have been developed to help researchers extract and visualize PubMed. Other researchers have built tools to rank search results, discover topics and relationships within search results, visualize search results, and improve user interaction with PubMed [12].

#### Text Mining PubMed

Jensen et al give a thorough overview of how PubMed can be harnessed for information extraction and entity recognition [6]. Natural language processing (NLP) techniques form one approach to mining the literature. Some researchers have used NLP techniques on PubMed to discover disease-gene associations [13], and others have used PubMed in concert with additional data sources to generate phenotypes [14]. Collier et al used NLP techniques in conjunction with association rule mining to discover phenotypes using PubMed [15]. However, none of these approaches have sought to use PubMed as a validation tool for data-driven phenotypes.

Co-occurrence analysis, which is what PheKnow-Cloud and PIVET are built on, is more widely used because it is simple to implement and interpret. Researchers have applied co-occurrence strategies to generate phenotypes. Some have performed co-occurrence analysis on PubMed to study links between diseases [16], which can be viewed as a simple type of phenotype discovery. Others have explored relationships between phenotypes and genotypes [17,18]. In contrast to this work, our approach uses phenotypes as the starting point and performs co-occurrence analysis over the PMC corpus as a means of assessing their validity. We assume these phenotypes were induced over other sources (eg, EHRs) and not from PMC. Co-occurrence analysis has the drawback of not being able to explicitly model the type of relationship that exists between two or more terms (eg, negative or positive). However, we require the terms within a phenotype be positively related to one another, which aligns with the findings of publication bias research.

Publication bias is the tendency for the academic publishing ecosystem (eg, researchers, reviewers, and editors) to submit and publish articles that show positive relationships between the entities being studied. The nonrandom omission of results that is not based on the quality of the methodology but on the direction of the results is a well-studied area of research and has been shown to have a negative effect on research in many cases [19-24]. In general, publication bias introduces risks to researchers and to the general public to which research is applied (via policies and treatment decisions). However, in PheKnow-Cloud and PIVET, this bias is a strength rather than a drawback because the current focus of PheKnow-Cloud and PIVET is on the presence of relationships within the user-supplied candidate phenotypes. Furthermore, as co-occurrence analysis does not attempt to infer information about the type of relationship or any causal information, the presence of publication bias allows for the assumption that when two phrases occur together, it may imply that a relationship exists [20,25,26].

#### PheKnow-Cloud Prototype

Phenotype evaluation via co-occurrence analysis of online articles was first introduced by Bridges et al [1]. Henderson and colleagues improved on the evaluation framework and developed a prototype tool implementing the approach called PheKnow-Cloud, which provided a Web interface for researchers and clinicians to interact with the technology [27]. We refer to the tool and framework introduced in these two works as PheKnow-Cloud. The input to the PheKnow-Cloud process is a set of potential phenotypes. Each phenotype consists of medical terms, which we refer to as phenotypic items, that are assumed to have been generated by an automatic high-throughput phenotyping process. PheKnow-Cloud builds evidence sets for batches of phenotypes based on co-occurrence analysis of the PubMed corpus (see [1,27] for details).

PheKnow-Cloud was developed as a proof-of-concept tool, and although it showed the PubMed corpus could be used to determine whether a phenotype was clinically valid, it had several drawbacks that PIVET addresses. One is the length of time the prototype method required to complete the analysis process; Table 1 compares the time that each method takes to perform each step. The computational bottlenecks for the prototype method are the co-occurrence generation and clinical relevance score analysis steps. The synonym generation step speed is determined by the number of requests that can be made to the NLM Medical Subject Headings (MeSH) database, which is an off-site system that places limits on the number of requests users can make in a given window of time. Overall, PIVET speeds up this process considerably. Another drawback of PheKnow-Cloud is that the clinical relevance scores for phenotypes are calculated only relative to all other phenotypes and must be used in a batch setting. In contrast, PIVET can analyze a single phenotype at a time, which makes it more flexible than PheKnow-Cloud. Finally, designating whether a candidate phenotype is clinically relevant or not is a manual process in PheKnow-Cloud. For PIVET, we built a classifier trained on a validated set of phenotypes. This classifier can be ported to other environments and can be used to automatically classify new, individual phenotypes.

**Table 1:** The time in seconds and (hours: minutes: seconds) each method used to complete task in phenotype generation process. All experiments were run on a machine with 3 AMD A6-5200 APU with Radeon (TM) HD Graphics processors, 8 GB of memory, 1 TB hard drive, running Ubuntu 14.04.5 LTS.

Task	PheKnow-Cloud	PIVET^a^
Synonym generation, seconds (hours:minutes:seconds)	7809 (02:10:09)	5948 (01:39:08)
Co-occurrence analysis, seconds (hours:minutes:seconds)	50,822 (14:07:02)	289 (00:04:59)
Lift analysis, seconds (hours:minutes:seconds)	2092 (00:34:52)	2 (00:00:02)
Total, seconds (hours:minutes:seconds)	60,723 (16:52:03)	6239 (01:43:59)

^a^PIVET: Phenotype Instance Verification and Evaluation Tool.

## Methods

### Methods Overview

**Figure 1:**
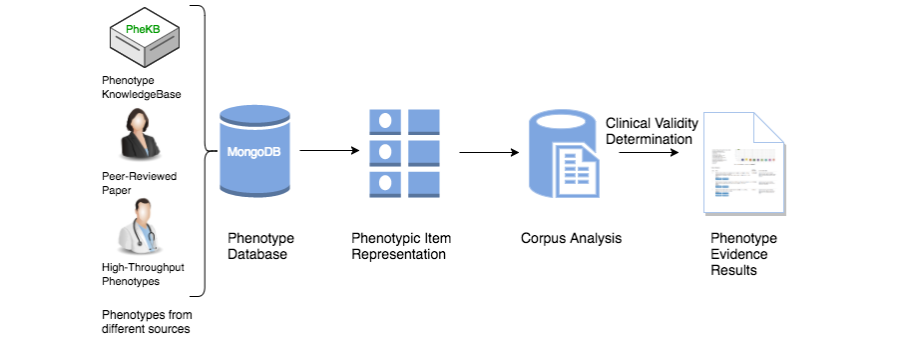
Phenotype Instance Verification and Evaluation Tool (PIVET) analysis process. Phenotypes are collected in standardized format in a MongoDB (ie, “phenotype database”). For a single phenotype, synonyms for each phenotypic item in a phenotype are generated using the National Library of Medicine (NLM) Medical Subject Headings (MeSH) database and ranked based on their similarity to the phenotypic item (ie, “phenotypic item representation”). Co-occurrence analysis is performed on PubMed using the synonyms generated in the previous step (ie, “corpus analysis”). Lift analysis is performed, clinical relevance scores are calculated, and a classifier classifies the phenotype as clinically relevant or not (ie, “clinical validity determination”). The results of the analysis of the phenotype are presented to the viewer (ie, “phenotype evidence results”).

In this section, we describe how PIVET performs co-occurrence analysis on an online corpus of publicly available journal articles to build evidence sets for phenotypes. This involves five components: (1) a database of phenotypes to analyze, (2) a database of the PubMed article corpus indexed by medical terms the articles contain, (3) an algorithm to generate and rank synonyms for the phenotypic items (phenotypic item representation), (4) a co-occurrence analysis module (corpus analysis), and (5) a clinical relevance scoring system (clinical validity determination). Figure 1 captures the PIVET workflow and the different components of the system. Both MongoDB (an open-source, document-based NoSQL database system) and MySQL (an open-source, relational database management system) are used to ensure consistency, durability, and efficiency.

### Phenotype Extraction and Storage

**Figure 2:**
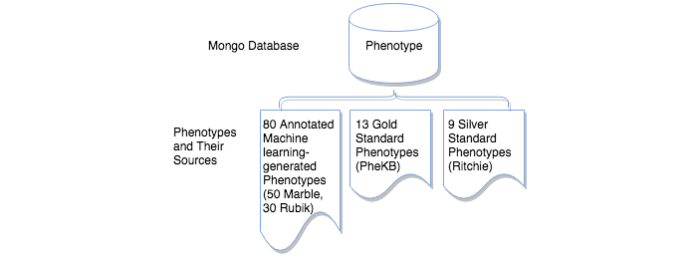
Database for storing phenotype information. The large cylinder at the top represents the phenotype database. The phenotype database consists of phenotypes (documents) extracted from three different sources (bottom). The first set of phenotypes, 80 in total, were generated by machine learning algorithms called Marble and Rubik and annotated for clinical relevance by 3 medical doctors. The second set of phenotypes, 13 in total, we refer to as gold standard phenotypes and come from Phenotype KnowledgeBase, an online repository of domain expert–developed phenotypes. The third set of phenotypes, 9 in total, we refer to as silver standard phenotypes and were derived by domain experts and extracted from a peer-reviewed journal article.

PIVET can be used to analyze phenotypes generated from a variety of methods. Every phenotype analyzed by PIVET is stored in a MongoDB using a standardized representation to ensure consistency. We also created a simple parser to ingest new phenotypes that are stored in JavaScript Object Notation (JSON). The choice of JSON will also facilitate the eventual integration with a Web platform where users can provide new phenotypes. We populate the phenotype database with phenotypes from different sources (Figure 2).

For our purposes, we collected a total of 102 phenotypes from the following sources: (1) two high-throughput phenotyping algorithms, (2) a catalog of algorithms from a collaborative database, and (3) a peer-reviewed paper. Each phenotype we extracted was either derived by domain experts or validated as clinically relevant by domain experts.

The phenotype database includes 80 domain expert–verified phenotypes generated using two unsupervised, nonnegative tensor factorization models to perform automated phenotyping [28,29]. These were subsequently annotated by a panel of domain experts, and they were the phenotypes used to validate PheKnow-Cloud. The two automatic methods, Rubik [29] and Marble [28], extracted 30 and 50 candidate phenotypes, respectively, from the diagnoses and medications of 7744 deidentified patients from Vanderbilt University Medical Center recorded over a 5-year observation period.

Each member of the panel assigned all phenotypes one of the following three labels: (1) *yes*, the candidate phenotype is clinically meaningful and therefore a phenotype; (2) *no*, the candidate phenotype is not clinically meaningful and therefore not a phenotype, or (3) *maybe*, the candidate phenotype is possibly clinically meaningful. Of the 80 combined Marble and Rubik phenotypes, the domain experts labeled 11 (14%, 11/80) as clinically meaningful, 62 (78%, 62/80) as possibly significant, and 7 (8%, 7/80) as not clinically meaningful. For the handful of phenotypes where the domain experts disagreed on the clinical relevance, the label that awarded the least amount of clinical significance was assigned. These annotated phenotypes were graciously shared by the authors of Rubik.

Additionally, the phenotype database includes two groups of domain expert–derived phenotypes. The first set, which we will refer to as the “gold standard” phenotypes, are from the Phenotype KnowledgeBase, an online phenotype knowledgebase that stores researchers’ collaborations of electronic algorithms of phenotypes [30]. Gold standard phenotypes are developed by panels of domain experts across multiple sites. We manually extracted 13 phenotypes that have been reviewed and finalized by the Electronic Medical Records and Genomics phenotype working group. The second set of domain expert–derived phenotypes, which we will refer to as “silver standard” phenotypes, are the group of validated phenotype algorithms published by Ritchie et al [31]. Silver standard phenotypes are developed by a panel of domain experts at a single site. Nine phenotypes were manually extracted from the article. This peer-reviewed paper is not part of the article corpus. In summary, the full set of 102 phenotypes collected over the three different sources consists of 80 machine learning–extracted phenotypes validated by domain experts, 13 gold standard phenotypes, and 9 silver standard phenotypes.

### PubMed Open Access Corpus

PIVET works by analyzing co-occurrences of phenotypic items within the PMC OA subset, an openly available online repository of medical articles, which constitutes roughly one-third of the total collection of articles in the PMC (over 1 million articles). The articles within the OA subset are copyright protected but have a flexible license concerning reuse. Trimmed down versions of the articles are stored in a MongoDB. We use the NoSQL database MongoDB because it is a document-based database without restrictive schema, ideal for storing articles that vary in content. Furthermore, MongoDB has been shown to outperform SQL-based databases in terms of read, write, and delete operations and scaling to larger datasets [32-34].

We limit the corpus in the database to those articles with attached MeSH terms; this amounts to 379,766 articles. MeSH is a hierarchical vocabulary curated by the NLM to index and catalog biomedical information [35]. There are 26,000 biomedical concepts or headings and over 200,000 supplementary concepts that form qualifiers for the headings. MeSH has two major benefits over the other existing ontologies. First, a large portion of the PubMed corpus has been manually annotated with MeSH labels. Expert indexers at the NLM assign MeSH terms to each article that best summarize the text. These terms are periodically reviewed and updated. We index the PMC database with the MeSH terms each article contains, and we represent each item in a phenotype with a set of MeSH terms, which is discussed in the next section. The index and phenotypic item representation combined with search optimization techniques described in the subsequent section speed up the co-occurrence analysis process considerably.

### Phenotypic Item Representation: Constructing Medical Subject Headings Synonym Sets

Once the phenotypes are stored in the database, the next step is to build representations for the terms within each phenotype, which we refer to as “phenotypic items.” Medical terms can have various synonyms (representations) across different articles. For example, the term “heart attack” can also be referred to as “cardiovascular stroke,” “myocardial infraction,” and “cardiogenic shock.” Thus, it is important to generate a list of synonyms for each phenotypic item to achieve high recall within the PubMed corpus. PheKnow-Cloud built representations for each phenotypic item from related terms and concepts found in the following medical ontologies: MeSH, Systemized Nomenclature of Medicine-Clinical Terms, and International Classification of Diseases-9 or -10. Further experiments indicated this approach can introduce noise into the representation. Instead, PIVET uses only MeSH terms to generate a phenotypic item representation for each phenotypic item with the following two-step process: (1) assign the most relevant MeSH term and (2) generate a ranked list of closely related MeSH terms.

To generate a candidate set of representations for a phenotypic item, PIVET first queries the NLM MeSH database using Biopython [11] with a cleaned version of the phenotypic item. The search returns a set of MeSH tree numbers. MeSH terms are formed into a hierarchical tree, where each MeSH term is assigned a node in the tree and labeled by a number. This number designates the MeSH term’s place in the hierarchy. For example, the tree number of “hypertension” is C14.907.489, which indicates that it is a child of the node C14.907 (“vascular diseases”). Vascular diseases, in turn, is a child of node C14 (“cardiovascular diseases”). Gathering nodes with the prefix C14.907.489 gives a set of possible synonyms for the original phenotypic item “hypertension.” Generally, this hierarchy gives a relatively straightforward method for finding synonyms and relevant concepts.

**Figure 3:**
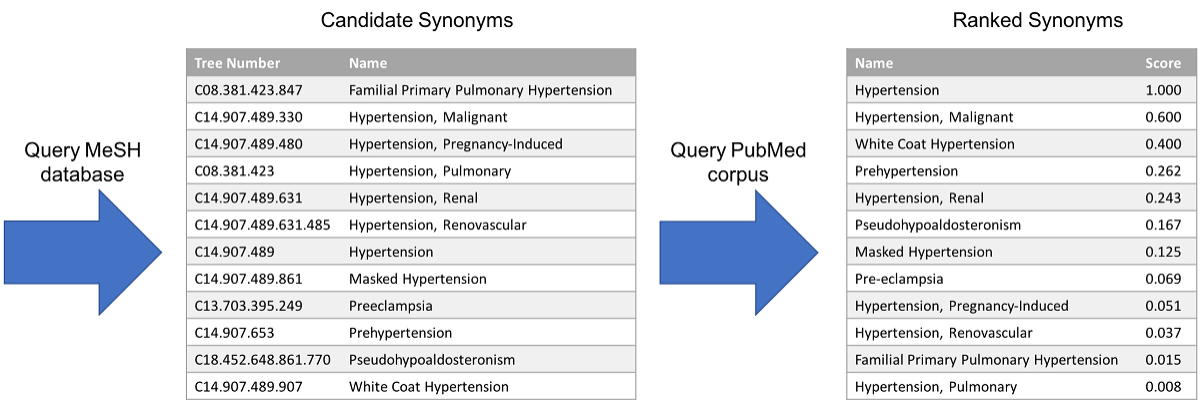
Synonym generation process for the term “hypertension.” First the National Library of Medicine (NLM) Medical Subject Headings (MeSH) database is queried with the term “hypertension,” which returns a list of candidate MeSH terms. From this query result, the “most relevant synonym” is determined through a process of string matching between the original queried term and the candidate synonyms. In this case, the most relevant synonym is “hypertension.” The candidate synonyms are then ranked based on the percentage overlap between PubMed articles that contain the MeSH term associated with the candidate synonym and the MeSH term of the most relevant synonym.

As the query does not rank the results (ie, it does not designate which tree number is most relevant to the search), it is necessary to identify the MeSH term that most closely matches the phenotypic item. For example, querying the phenotypic item “hypertension” returns the tree numbers that map to the natural language headings: “hypertension, malignant”; “hypertension, portal”; “hypertension, pulmonary”; “hypertension, renal”; “hypertension”; “masked hypertension”; “prehypertension”; etc (shown in Figure 3). PIVET designates the “most relevant synonym” for the original phenotypic item by finding the natural language heading associated with each of the tree numbers that most closely matches the original phenotypic item. Specifically, for each natural language heading or synonym, PIVET forms a set where each element is a word of the synonym and then finds size of the intersection between the set and the original cleaned item, which has also been turned into a set. It also records the size difference between the two sets. For example, the phenotypic item “hypertension” and candidate synonym “hypertension, malignant” have an intersection of length one (ie, “hypertension”) and a size difference of 1. However, PIVET would assign “hypertension” as the most relevant synonym because it has an intersection of size 1 and a set size difference of 0 with the original phenotypic item. In the event of a tie, the algorithm designates the tied candidate synonyms as the most relevant synonyms and builds the synonym sets for each.

The remaining synonyms are then ranked based on the percentage overlap between each candidate synonym and the most relevant synonym in our PubMed OA corpus. The percentage overlap, calculated as the number of times the candidate synonym appears with the most relevant synonym divided by the number of times the candidate synonym appears overall, serves as the relevance score to rank each synonym. The ranked list is then used to adjust the number of synonyms. An example of a ranked synonym set can be seen in Figure 3.

### Corpus Analysis

The aim of the corpus analysis step is to gauge the strength of the relationship between items in a phenotype. However, it is unlikely all items in a phenotype will appear together, so instead, PIVET searches the corpus for occurrences of subsets of the phenotypic items (represented by their phenotypic item MeSH synonym sets as described in the last section). Through experimentation, we found only a small fraction of subsets of any phenotype occur in the article corpus. This means it is inefficient as well as computationally infeasible for even moderately sized phenotypes to look for all possible subsets (ie, the power set in this case has 2^|S| * n_1_ * n_2_ * …* n_|S|_ elements, where |S| is the cardinality of the phenotype and is the synonym set size for phenotypic item *i*).

Moreover, as the size of the subset increases, the likelihood of all the terms appearing in any given article diminishes. Therefore, it is not necessary to enumerate all the possible subsets.

Using this observation, we implement an algorithm inspired by the string-matching Aho-Corasick algorithm to search the space effectively [36], an approach also made popular by the a priori algorithm for finding association rules in data mining. We sketch the algorithm with a set comprised terms A, B, C, and D that we assume all occur individually in the corpus. We observe that if terms A and B, comprising a tuple (A,B), do not co-occur in any article together, then any larger subset also containing these two terms will necessarily have zero counts (eg, [A,B,C], [A,B,D], and [A,B,C,D]). As a result, only nonzero (feasible) co-occurrence subsets need to be expanded. A key insight for efficient expansion of an existing co-occurrence subset with nonzero counts is to join it with the associated tuple pairs with one overlapping term that have nonzero counts. For example, if the only nonzero tuple pairs are (A,C), (A,D), (B,C), (B,D), and (C,D), then the possible tuples with cardinality 3 are (A,C,D) and (B,C,D). As increasing the cardinality size of the tuple is equivalent to a join operation in a SQL database, PIVET uses MySQL to implement this portion of the analysis. After constructing the query tuples of MeSH terms in MySQL, PIVET then counts the number of articles where each tuple appears.

Additionally, we set a few more restrictions on the subset queries to make them even more efficient. For one, each subset is constructed using “different” phenotypic items to avoid arbitrary inflation of counts. If two or more phenotypic items contain identical MeSH synonym sets, a “super” phenotypic item is formed (eg, “tuberculosis of adrenal glands” and “tuberculosis of adrenal glands, bacteriological or histological examination not done” are merged together). In addition, terms for the same phenotypic item (eg, all MeSH terms associated with “myocardial infraction”) are never paired with one other.

Given these tuple co-occurrence counts, the next step is to map the co-occurring subsets of phenotypic synonyms back to their phenotypic items. For example, if the synonym set for the phenotypic item “attention deficit disorder” contains two synonym terms “attention deficit and disruptive behavior disorders” and “attention deficit disorder with hyperactivity,” then any tuple of cardinality 1 with either of these terms is collected, and the sum of the co-occurrences is then designated as the number of times the phenotypic item “attention deficient disorder” occurred. The aggregated co-occurrence counts for all the nonzero subsets of the phenotypic items are then used to calculate the clinical relevance scores for the phenotype.

### Clinical Validity Determination

**Figure 4:**
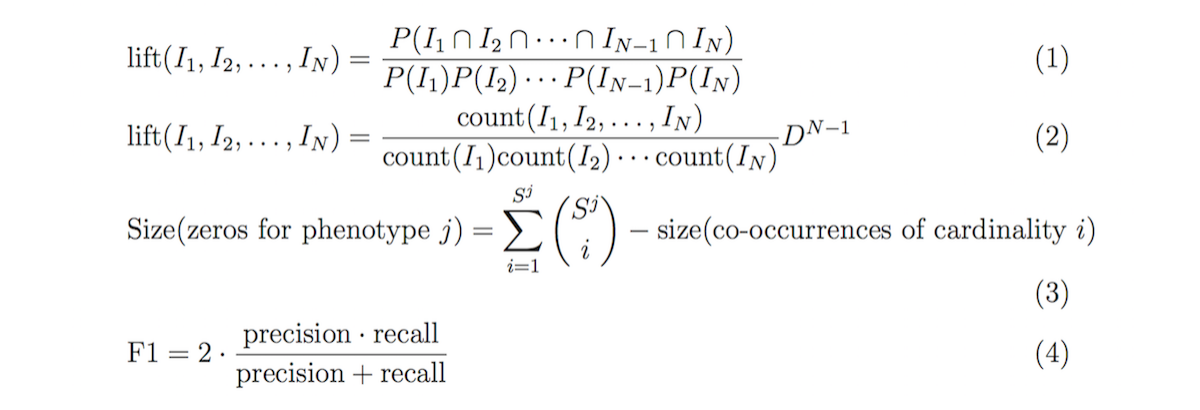
Lift, number of zeros, and F1 equations.

PIVET uses a two-step process to calculate the clinical relevance score: (1) obtain the *lift* (see below) for each co-occurring subset of phenotypic items and (2) classify the relevance of the phenotype based on features derived from the previous step. As in PheKnow-Cloud, PIVET uses lift to evaluate the strength of the relationship between the items in a phenotype. Given items I_1_, I_2_, …, I_N_, lift is defined in equation 1 in Figure 4.

Lift is a widely used metric to measure the statistical independence of objects [37]. A lift of greater than 1 suggests a nonrandom relationship. Although there are many metrics (eg, support, gain, certainty, confidence, and coverage) that can help assess the plausibility of relationships between objects, lift has the benefit of being symmetric (ie, lift[A,B]=lift[B,A]), and therefore, the order of the objects does not matter [38]. Another metric called leverage also has this symmetric property. However, unlike leverage, lift is not impaired by the “rare item problem,” which refers to the property of a metric excluding objects that appear infrequently [39]. In the OA corpus, phenotypic items appear infrequently, so it is especially important to use a metric that does not suffer from the rare item problem. In PIVET, the lift calculation entails dividing the percentage of times items appear together in the corpus by the product of percentages of times each item appears individually in the corpus, which can be rewritten as equation 2 in Figure 4, where count(A) is the number of articles in the corpus that contain the set A, and D is the number of documents in the corpus.

It was observed in PheKnow-Cloud that the lift increases exponentially with the size of the co-occurrence set [1]. This is consistent with equation 2. For example, if a set of six items appears together then the fraction of counts will be multiplied by the size of the corpus to the fifth power. These lifts of larger co-occurring subsets drown out the lifts of smaller-sized subsets, which is not necessarily desirable. Thus, we must “normalize” the cardinality of co-occurrence sets. To this end, PheKnow-Cloud calculated the lift for any subset that occurred in the corpus without regard to whether the subset occurred in a phenotype, separated the lifts by the cardinality of the subsets, computed the SDs above the median within that cardinality, aggregated all the SDs above the median values back into the respective phenotypes, and averaged the SD values for each phenotype. This average served as the “clinical relevance score” for that phenotype. This implies that the relevance score will vary depending on the phenotype corpus, as phenotype scores are relative to other candidate phenotypes.

PIVET mitigates this issue inherent to PheKnow-Cloud normalization by including the number of tuples with zero co-occurrences. The number of subsets that had zero occurrences in the corpus is calculated using a simple combinatorial formula as shown in equation 3 in Figure 4, where S^j is the number of phenotypic items in phenotype j.

Including the zero occurrence counts for each cardinality pulls down the overall lift of the larger items (as it is improbable that large subsets of the phenotype will occur) and thus mitigates the impact of larger co-occurring subsets. Consequently, PIVET avoids the need to pool the phenotypic items across all the phenotypes and avoids unnecessary co-occurrence queries for tuples that do not occur in a phenotype. Perhaps more importantly, this implies that the relevance score is decoupled from the phenotype corpus and can be computed independently for a given phenotype.

The final step in the process is to classify the relevance of the phenotype. We compared four separate classification models: logistic regression, logistic regression with least absolute shrinkage and selection operator (lasso), ridge logistic regression, and k-nearest neighbors (k-NN) on the entire phenotype corpus to predict clinically significant vs not clinically significant. Gold and silver standard phenotypes are denoted as clinically significant because of their relatively small numbers.

The features we use are lift mean, lift median, and lift SD for each individual cardinality from 1, 2, 3, and 4 (12 features). We also include the overall lift mean, median, and SD (3 features) and the average cardinality of subsets of the phenotype with nonzero co-occurrences (16 features in total). Model-specific parameters (ie, K for k-NN and the regularization parameter for ridge and lasso) are chosen based on the best area under receiver operating characteristic via five-fold cross-validation.

In summary, the PIVET lift analysis differs from that performed by PheKnow-Cloud in two key ways. First, we eliminate the need to pool the lifts across the entire phenotype corpus, which means that phenotypes can be analyzed on an individual basis. Second, we introduce classification models to determine relevance based on lift-based features, removing the need to perform an exhaustive search to determine the clinical relevancy threshold.

## Results

### Results Overview

PIVET is evaluated using two different methods. The first compares the new framework with its predecessor, PheKnow-Cloud, on the set of phenotypes PheKnow-Cloud examined. Differences in computation time, synonym generation, and clinical relevance scores are quantitatively and qualitatively examined. This comparison shows that PIVET delivers clinical relevance determination performance comparable with that of PheKnow-Cloud in a fraction of the time. Furthermore, PIVET’s performance justifies shifting from the old to the new framework.

In the second set of experiments, we demonstrate the full PIVET framework on the combined set of machine learning–generated phenotypes, gold standard phenotypes, and silver standard phenotypes. This experiment and discussion show how PIVET’s classification method can be used to identify clinically relevant phenotypes from the pool of possibly clinically relevant phenotypes.

### PheKnow-Cloud Versus Phenotype Instance Verification and Evaluation Tool Comparison

#### Phenotypic Item Representation

A subset comprising one-quarter of the PMC OA corpus is used to compare our framework’s use of MeSH terms for the phenotypic item synonym sets with PheKnow-Cloud’s phenotypic item synonym sets. This subset is identical to the one used in the original evaluation of Pheknow-Cloud (see [1] for more details regarding the construction of the dataset). We limit this subset to articles with MeSH terms, which results in a corpus of articles that comprises 7.85% of the PMC OA subset (94,673/1,206,506). We restrict the phenotypes in question to the 80 domain expert–verified, machine learning–generated phenotypes used in the original papers [1,27]. Table 2 shows the clinical validity annotations of these 80 phenotypes.

PIVET takes less than 2 hours to evaluate 80 phenotypes on the 8% PMC OA subset; PheKnow-Cloud required 17 hours for the same phenotypes. The breakdown of the computation time for the major components of the two frameworks is shown in Table 1. The phenotypic item representation process time is roughly the same for both PIVET and PheKnow-Cloud, and querying the NLM MeSH database remains the bottleneck. However, PIVET is 170 and 35 times faster for the corpus analysis and clinical relevance determination steps, respectively. Not only does PIVET provide an overall speedup of 10 times on the same article corpus, but the entire process does not need to be repeated to analyze new phenotypes.

**Table 2:** Counts of the 80 machine learning–generated phenotypes by clinical relevance annotation category.

Domain expert annotation category	Count, n (%)
Clinically significant	11 (14)
Possibly clinically significant	62 (78)
Not clinically significant	7 (8)

**Figure 5:**
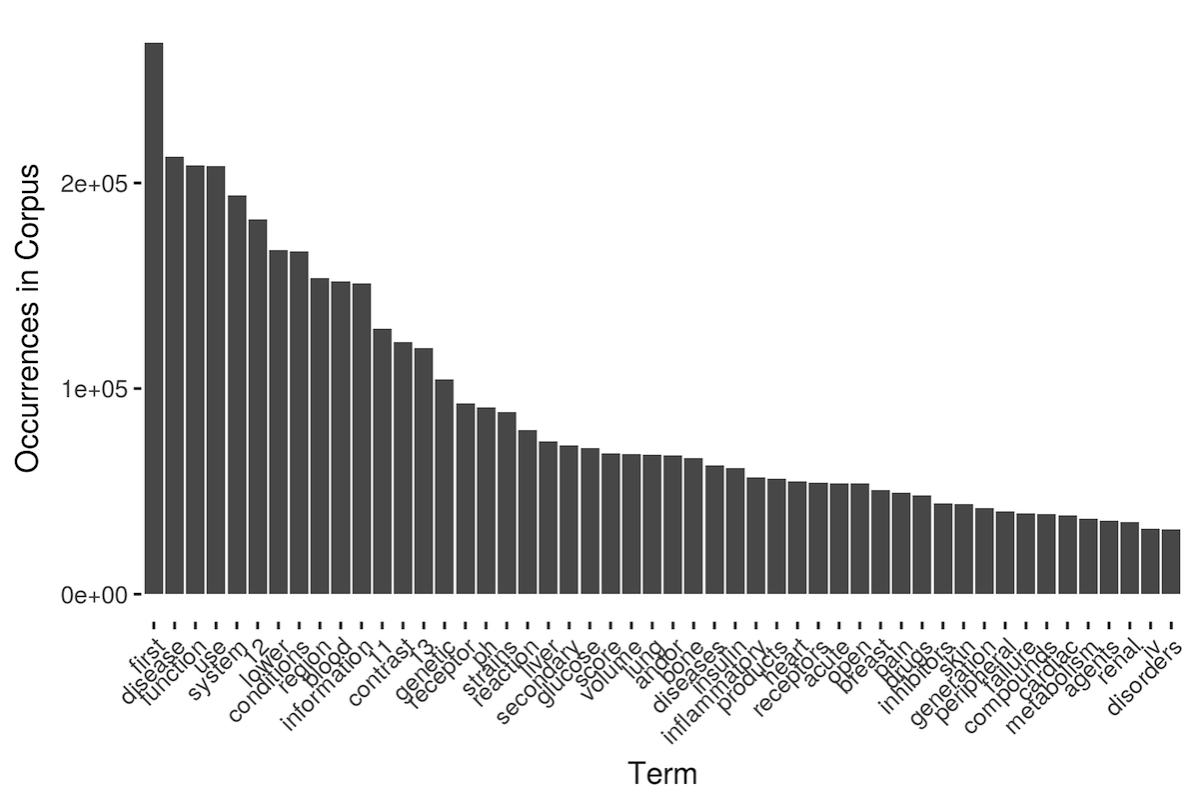
Most common synonyms found in corpus using PheKnow-Cloud synonym generation process.

As discussed in an earlier section, the phenotypic item representation is different between the two frameworks. PIVET uses sets of MeSH terms to represent each phenotypic item, whereas PheKnow-Cloud’s representative synonym sets are built from several ontologies that include the MeSH terms. Overall, PIVET finds more descriptive, discriminative, and possibly more interpretable representations of phenotypic items, whereas PheKnow-Cloud’s synonym sets produced a sizeable number of less descriptive words in comparison. Figure 5 shows the top 50 PheKnow-Cloud-generated synonyms that were found in the corpus. Although PheKnow-Cloud excludes the first 30 most common terms from its co-occurrence analysis, the remaining 20 words are not discriminative. For example, the word “diseases” is associated with many of the phenotypic items but is too generic to be a meaningful representation of the items.

Further qualitative evidence of the nonspecific nature of the synonym sets produced by PheKnow-Cloud can be found by consideration of examples. Table 3 shows the synonyms for the phenotypic item “unspecified chest pain.” Under the PheKnow-Cloud framework, although discriminative terms such as “unspecified chest pain” and “chest pain” are present in the synonym set, the terms “pain,” “chest,” and “unspecified” are words that will be present in many articles that do not actually refer to “unspecified chest pain.” In contrast, under the PIVET framework, the MeSH term for “unspecified chest pain” is “chest pain,” which while less specific than the original term, has the advantage that it will only be found in articles that mention chest pain.

**Table 3:** Comparison of representation of the phenotypic item “unspecified chest pain” generated by PheKnow-Cloud (left column) and Phenotype Instance Verification and Evaluation Tool (PIVET; right column).

PheKnow-Cloud (synonyms)	PIVET^a^ (MeSH^b^ terms)
Unspecified chest pain	Chest pain
Chest pain	—
Unspecified chest	—
Pain	—
Chest	—
Unspecified	—

^a^PIVET: Phenotype Instance Verification and Evaluation Tool.

^b^MeSH: Medical Subject Headings.

In some cases, the synonym sets are reasonable representations of the item and similar for both frameworks. For example, PIVET and PheKnow-Cloud can capture the meaning of the phenotypic item “laxatives” (shown in Table 4). PheKnow-Cloud extracts synonyms that are close literal matches to the phenotypic item or specific kinds of laxatives. Similarly, PIVET finds a MeSH term that is an exact match to the phenotypic item and a specific example of the phenotypic item. When looking through the corpus for occurrences of the original term “laxatives,” both frameworks should recover mentions of the original term.

#### Clinical Validity Determination

Next, we examine how PIVET’s phenotype representation compares with that of PheKnow-Cloud in terms of identifying clinically relevant phenotypes. To do this, we instrumented PIVET to record co-occurrences in the same manner as PheKnow-Cloud. Table 5 summarizes the number of articles that are found under each framework. Although the PIVET MeSH representation identifies significantly fewer articles from the corpus, the articles have an 85% overlap with PheKnow-Cloud articles. In conjunction with Figure 5 and Table 2, the results suggest that not all of the PheKnow-Cloud articles are relevant or directly related to the phenotypic item. Thus, PIVET synonym sets may result in higher precision.

**Figure 6:**
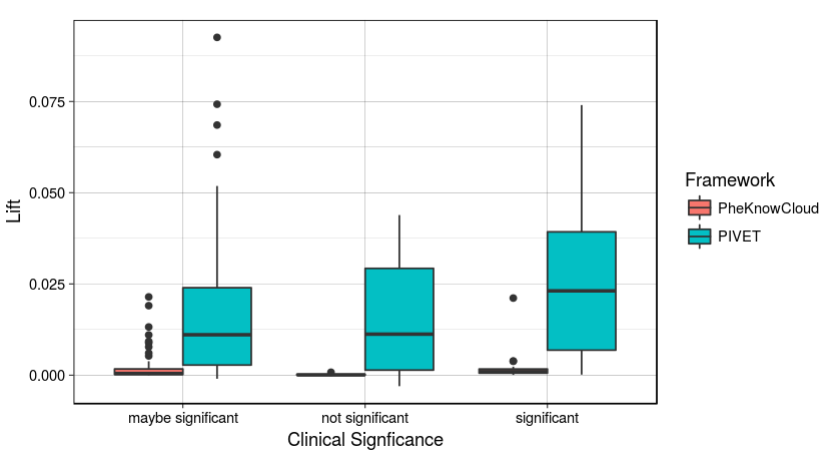
Normalized lift comparison between Phenotype Instance Verification and Evaluation Tool (PIVET) and PheKnow-Cloud. Normalized lift is calculated as follows: the lift for any subset of phenotypic items that occurred in the corpus without regard to whether the subset occurred in a phenotype is calculated. Then the lifts are separated by the cardinality of the subsets, and the standard deviations above the median within that cardinality is computed (ie, this is the normalized lift). The boxplot depicts the normalized lift for the subsets that appeared in each type (ie, “maybe significant,” “not significant,” and “significant”) of phenotype.

Finally, we compared the two frameworks’ ability to discriminate between clinically significant and not significant phenotypes using the process PheKnow-Cloud used. To do this, we first calculated the normalized lift for all the phenotypes using the synonyms sets generated by PheKnow-Cloud and PIVET. Figure 6 plots the pooled normalized lift values for the 80 phenotypes based on the annotated significance level. As we saw in the PheKnow-Cloud framework, under the PIVET representation, the distributions of normalized lift between significant and not significant phenotypes are not identical, which indicates that lift scores can be used to discriminate between significant and not significant phenotypes.

In the final step, we calculated clinical validity scores for each phenotype by taking the average of the normalized lift scores in each phenotype. An exhaustive search was performed on the clinical validity scores to determine the boundaries for PIVET and PheKnow-Cloud, which was the method used in PheKnow-Cloud that maximized the F1 score. F1 is computed as equation 4 in Figure 4.

We obtained an F1 score of 0.85 and 0.89 for PIVET and PheKnow-Cloud, respectively. Although the predictive performance of PIVET is slightly lower than that of PheKnow-Cloud, the performance loss is negligible when compared with the total run time of each framework (Table 1) on 8% of the PMC OA subset. Moreover, by mapping directly to MeSH terms, PIVET can leverage the “automatic” assignment of MeSH terms for all articles and can have a higher probability of capturing appearances of the original phenotypic item in the corpus.

**Table 4:** Comparison of representation of the phenotypic item “laxatives” generated by PheKnow-Cloud (left column) and Phenotype Instance Verification and Evaluation Tool (PIVET; right column).

PheKnow-Cloud (synonyms)	PIVET^a^ (MeSH^b^ terms)
Laxatives	Laxatives
Laxatives pharmacological action	Senna extract
Psyllium	—
Senna	—
Senna extract	—

^a^PIVET: Phenotype Instance Verification and Evaluation Tool.

^b^MeSH: Medical Subject Headings.

**Table 5:** Number of articles that each framework’s synonym generation process found.

Synonym type	Number of articles
PIVET^a^	28,068
PheKnow-Cloud	79,786
PIVET and PheKnow-Cloud	23,901

^a^PIVET: Phenotype Instance Verification and Evaluation Tool.

### Phenotype Instance Verification and Evaluation Tool

In the first set of experiments, we demonstrated PIVET’s synonym generation process results in discriminative performance comparable with that of PheKnow-Cloud in a fraction of the time. In the second set of experiments, we use PIVET’s full framework (Figure 1) to predict which phenotypes are clinically valid and show how PIVET can be used to examine phenotypes that are possibly clinically valid.

#### Corpus Analysis: Classification Score Evaluation

We evaluated the ability of the PIVET classification system to identify clinically significant phenotypes. The entire phenotype corpus, including the gold and silver standard phenotypes, were analyzed using the entire PMC OA corpus. There is ambiguity regarding the “possibly significant” Marble and Rubik phenotypes, and they were therefore excluded from the training set. Thus, a total of 45 phenotypes were used to build the classifier, with 7 annotated as not significant.

**Figure 7:**
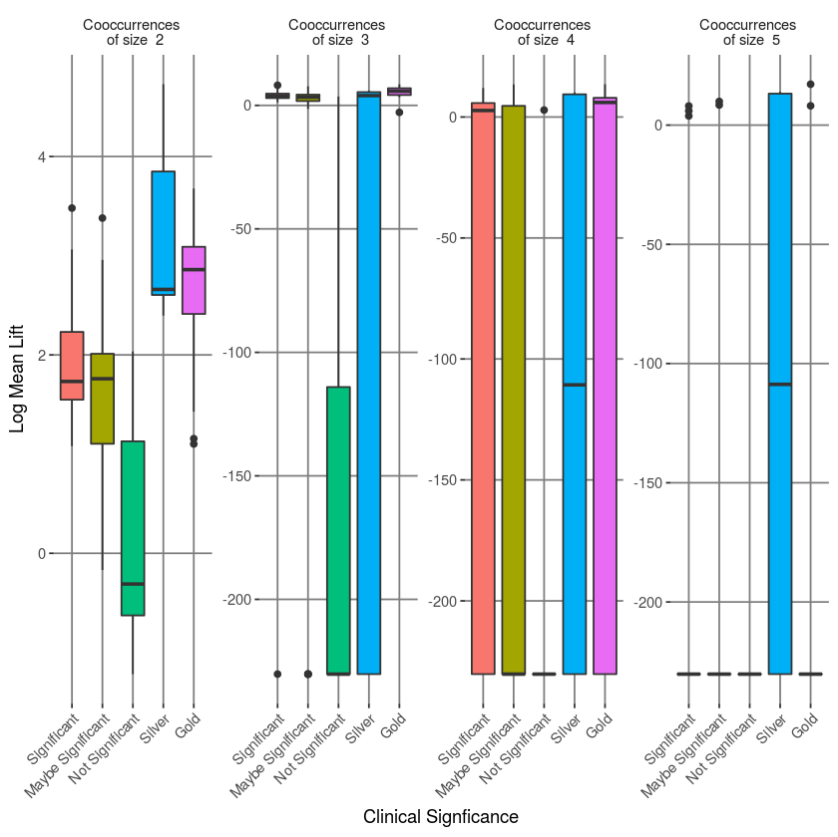
Log mean lift for co-occurrences of sizes 2, 3, 4, and 5 for each type of phenotype.

The diversity of the phenotypes in our corpus yielded phenotypes that contained anywhere from 3 to 63 phenotypic items. The size of the phenotype sets impacted the cardinality of the nonzero co-occurrence tuples; thus, we limited the lift summary features to only include tuples up to 4 (the average across the phenotype corpus). Figure 7 illustrates the differences in the mean lift values between the various categories, with the gold and silver standard separated from the clinically significant group. The results show that the phenotypes that are clinically significant exhibited a higher (more positive) distribution in lift mean compared with the nonsignificant phenotypes. Moreover, for co-occurrence cardinality less than 5, gold standard phenotypes generally had a higher lift. The figure suggests it is suitable to use the mean lift of tuples of cardinalities 2, 3, and 4 as individual features to distinguish the clinical significance of a phenotype.

**Figure 8:**
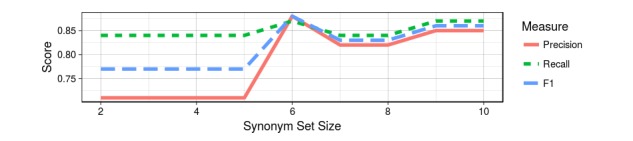
Classification scores for different sizes of synonyms using the Phenotype Instance Verification and Evaluation Tool (PIVET) framework.

Next, we used logistic regression to analyze the effect of the size of the synonym set. For each synonym set size ranging from 2 to 10, we used five-fold cross-validation to examine how the size of the synonym set generalizes to an unseen dataset for different metrics. Figure 8 plots the average precision, recall, and F1 score as a function of the synonym set size. The figure shows significant increases for all three metrics at synonym size 6, at which point an F1 score of 0.89, recall rate of 0.89, and a precision score of 0.88 are achieved. On the basis of these results, we used six synonyms for each phenotypic item for the remaining analysis.

We repeated the classification process using four models (logistic regression, k-NN, logistic regression with lasso, and ridge-regularized logistic regression) with six MeSH term synonyms for each phenotypic item. The results are shown in Table 6. Of the four classification models, ridge regression achieved the highest F1 score of 0.91 and an Area Under the Receiver Operating Curve score of 0.60. On the basis of these results, we use ridge regression as our classification model for the remaining results. Incorporating a classification model into the framework is an improvement over PheKnow-Cloud, which depended on an exhaustive search to obtain a boundary between clinically relevant and not clinically relevant phenotypes.

**Table 6:** Performance metrics for classification task to identify clinically relevant phenotypes using synonym sets of size 6.

Metric	Logistic regression	K-nearest neighbors	Lasso	Ridge regression
Area Under the Receiver Operating Curve	0.79	0.72	0.33	0.60
F-1	0.87	0.90	0.77	0.91

**Table 7:** Diagnoses and medications for candidate phenotypes along with domain expert annotations, classification score, and lift for two possibly significant phenotypes with high (top two rows) and low (bottom two rows) classification scores.

Diagnoses	Medications	Comment	Score	Lift
Hypotension, heart failure, cardiac dysrhythmias, unspecified chest pain, ischemic heart disease, hypertension, cardiomyopathy	Statins, proton pump inhibitors, gabapentin, noncardioselective beta blockers, sodium, group v antiarrhythmics, potassium-sparing diuretics	The arrhythmic heart patient	1	317.380
Disorders of fluid, electrolyte, and acid-base balance; other and unspecified anemias; hypertensive chronic kidney disease; hypertension; diabetes mellitus; type 2; other disorders of kidney and ureter; chronic kidney disease	Antiadrenergic agents, centrally acting, angiotensin receptor blockers, angiotensin converting enzyme inhibitors, selective immunosuppressants, loop diuretics, gabapentin	Heading toward dialysis	0.999	24683.383
Volume depletion; dehydration, nausea, or vomiting; hypopotassemia; abdominal pain	Heparins, antihistamines, 5HT3 receptor antagonists, minerals and electrolytes, narcotic analgesic combinations, proton pump inhibitors	Gastroenteritis	0.418	0.270
Disorders of fluid, electrolyte, and acid-base balance; other diseases of lung; hypotension; pleurisy, atelectasis, and pulmonary collapse; unspecified chest pain; other disorders of the kidney and ureter	Anticholinergic bronchodilators, loop diuretics	Lung diseases?	0.417	0.509

#### Clinical Validity Determination: Phenotype Instance Verification and Evaluation Tool Analysis of Possibly Clinically Significant Phenotypes

We demonstrate the potential of using PIVET to annotate phenotypes by examining the 62 “possibly clinically significant” phenotypes in our phenotype dataset. Using the PIVET classification ridge model, we predicted the clinical relevance scores of these ambiguous phenotypes. Table 7 shows the two extremes based on the averaged prediction score: phenotypes with the highest probability of being “clinically significant” (top two rows) and phenotypes with the lowest probability of being “clinically significant” (bottom two rows), as well as the annotator’s comment on the phenotype and the average lift calculated by PIVET. The prediction scores seem to reflect the annotator’s certainty, as the lowest prediction score is associated with a question mark, whereas the top two scoring phenotypes seem to capture a relevant concept. The results underscore the potential of PIVET system to help resolve uncertainties.

## Discussion

### Principal Findings

Several automated, high-throughput phenotype methods have been proposed to help clinicians quickly characterize and understand vast amounts of healthcare data. However, the potential for computational phenotyping to help physicians reason about patient populations will only be realized if the phenotypes generated are clinically meaningful. To increase the utility of such data-driven phenotype discovery, some measure of inferred clinical meaningfulness should be reported to help clinicians sort the signals from the noise. We developed PIVET to meet this need. PIVET generates evidence sets and clinical relevance scores for data-driven candidate phenotypes using the literature available in PubMed, a large online repository of biomedical articles.

We compared our framework with PheKnow-Cloud, its predecessor, and showed that PIVET improves the run time dramatically. In addition to scaling up to the entire PMC OA corpus, PIVET can analyze phenotypes individually and automatically assign clinical relevance scores that are independent of the other phenotypes in the corpus. Furthermore, there was anecdotal evidence that the PIVET synonym generation process was more discriminative and meaningful than its PheKnow-Cloud counterpart. In the future, one goal is to make PIVET available to researchers and clinicians. To this end, we plan to deploy a live version of the phenotype parser that users can interact with via a REST API and receive phenotype JSON files in return. We are currently investigating the best way to release PIVET for general use.

### Possible Use Cases

For researchers developing models and algorithms to automatically extract phenotypes from EHRs without supervision, all phenotypes are possibly clinically significant before they have been validated. We envision PIVET being used by researchers to gain understanding into the phenotypes they have extracted. Outside a machine learning setting, there are several potential uses for PIVET. For example, a pharmaceutical company may uncover a potentially interesting pathway analysis or phenotype, and they can use PIVET to identify all the articles that have been previously published on the subject, as well as PIVET’s clinical validity determination to decide if the pathway is worth pursuing and how much it can be trusted. Similarly, in a healthcare setting, a clinician could encounter an interesting group of patients and use PIVET to explore what pathways have been discovered with relation the set of patient characteristics. As in the pharmaceutical setting, PIVET’s ability to deliver a clinical validity determination, as well as generate a body of evidence in the form relevant articles, can help clinicians reason about the patterns they encounter on a daily basis.

### Limitations

One possible way to improve PIVET is to include more phenotypes when training the classifier. We continue to gather additional domain expert annotated phenotypes to include in the framework. One limitation of this analysis was that all the gold and silver standard phenotypes were combined with the domain expert–labeled examples for classification purposes. As we continue to gather more gold and silver phenotypes, we plan to refine the classification process by incorporating this “annotation quality” information. We also plan to test new sets of features that incorporate interaction between the lift statistics and to examine different metrics for evaluating the clinical significance of candidate phenotypes.

## Abbreviations

EHR: electronic health record
JSON: JavaScript Object Notation
k-NN: k-nearest neighbors
lasso: least absolute shrinkage and selection operator
MeSH: Medical Subject Headings
NLM: National Library of Medicine
NLP: natural language processing
OA: open access
PIVET: Phenotype Instance Verification and Evaluation Tool
PMC: PubMed Central
